# Association between gene polymorphisms in the cyclophosphamide metabolism pathway with complications after haploidentical hematopoietic stem cell transplantation

**DOI:** 10.3389/fimmu.2022.1002959

**Published:** 2022-09-23

**Authors:** Paula Muñiz, Cristina Andrés-Zayas, Diego Carbonell, María Chicano, Rebeca Bailén, Gillen Oarbeascoa, Julia Suárez-González, Ignacio Gómez Centurión, Nieves Dorado, David Gallardo, Javier Anguita, Mi Kwon, Jose L. Díez-Martín, Carolina Martínez-Laperche, Ismael Buño

**Affiliations:** ^1^ Department of Hematology, Gregorio Marañón General University Hospital (HGUGM), Madrid, Spain; ^2^ Gregorio Marañón Health Research Institute (IiSGM), Madrid, Spain; ^3^ Genomics Unit, Gregorio Marañón General University Hospital (HGUGM), Madrid, Spain; ^4^ Department of Hematology, Instituto Catalan de Oncología Hospital Josep Trueta, Girona, Spain; ^5^ Department of Medicine, School of Medicine, Complutense University of Madrid, Madrid, Spain; ^6^ Department of Cell Biology, School of Medicine, Complutense University of Madrid, Madrid, Spain

**Keywords:** polymorphisms, cyclophosphamide metabolism genes, haploidentical stem cell transplantation, Graf-versus-host disease (GVHD) prophylaxis, post-transplant complications

## Abstract

Allogeneic hematopoietic stem cell transplantation (allo-HSCT) is a curative treatment for patients with hematologic malignances. Haploidentical HSCT (Haplo-HSCT) is an alternative option for patients who do not have an HLA-matched donor. The use of post-transplantation high dose cyclophosphamide (PT-Cy) is commonly employed for graft-versus-host disease (GVHD) prophylaxis in haplo-HSCT. Cyclophosphamide (Cy) is an alkylating agent with antineoplastic and immunosuppressive activity, whose bioactivation requires the activity of polymorphic enzymes in the liver to produce phosphoramide mustard, which is a DNA alkylating agent. To identify polymorphisms in the genes of Cy metabolism and correlate them with post-HSCT complications [GVHD, sinusoidal obstruction syndrome (SOS), hemorrhagic cystitis (HC) and transplant-related mortality (TRM)], we designed a custom next-generation sequencing panel with Cy metabolism enzymes. We analyzed 182 patients treated with haplo-HSCT with PT-Cy from 2007 to 2019, detecting 40 variants in 11 Cy metabolism genes. Polymorphisms in CYP2B6, a major enzyme involved in Cy activation, were associated with decreased activity of this enzyme and a higher risk of Graf-versus-host disease (GVHD). Variants in other activation enzymes (CYP2A6, CYP2C8, CYP2C9, CYP2C19) lead to decreased enzyme activity and were associated with GVHD. Polymorphisms in detoxification genes such as glutathione S-transferases decreased the ability to detoxify cyclophosphamide metabolites due to lower enzyme activity, which leads to increased amounts of toxic metabolites and the development of III-IV acute GVHD. GSMT1*0 a single nucleotide polymorphism previously recognized as a risk factor for SOS was associated with a higher risk of SOS. We conclude that polymorphisms of genes involved in the metabolism of cyclophosphamide in our series are associated with severe grades of GVHD and toxicities (SOS and TRM) after haplo-HSCT and could be used to improve the clinical management of transplanted patients.

## Introduction

Allogeneic hematopoietic stem cell transplantation (allo-HSCT) has been established as a potentially curative treatment for patients with hematological malignancies. The beneficial graft-*versus*-leukemia effect, however, is associated with graft-*versus*-host disease (GVHD) and subsequent transplant-related mortality (TRM). Several strategies are applied to prevent GVHD, and post-transplantation high-dose cyclophosphamide (PT-Cy) is one of the most commonly employed in haploidentical HSCT (haplo-HSCT). PT-Cy eliminates expanding alloreactive T cells without affecting stem cells. This prophylactic strategy can prevent the onset of GVHD while maintaining immune reconstitution and controlling relapses (1).

Cyclophosphamide (Cy) is an alkylating agent used for the treatment of hematological malignancies and solid tumors as well as an immunosuppressive agent that affects T cells and B cells (2). Cy is an inactive prodrug whose bioactivation requires the activity of phase I metabolism cytochrome P450 (CYP450) enzymes in the liver, where high levels of drug-metabolizing CYP450 enzymes are expressed (3).

**Figure 1:**
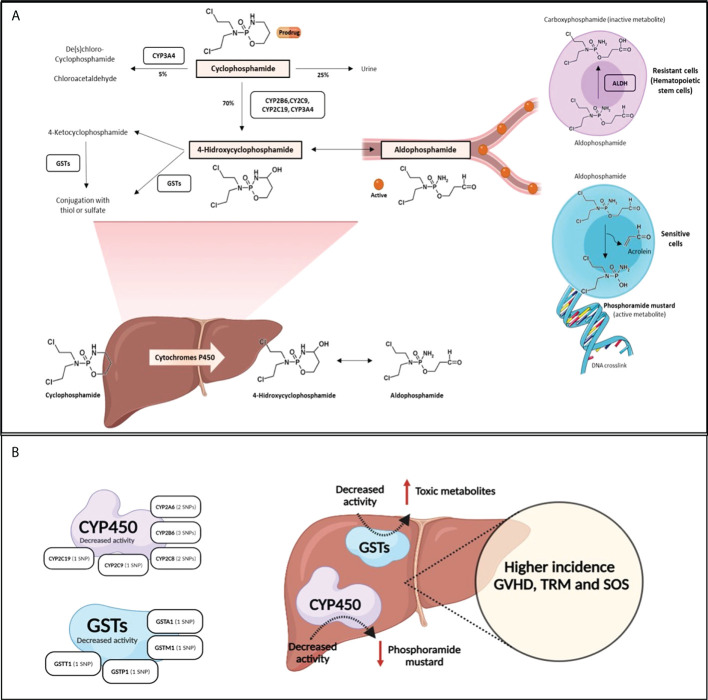
The metabolic pathway of cyclophosphamide. **(A)**. Cy (prodrug) is activated by the hepatic CYP450 to 4-hydroxycyclophosphamide, which stays in equilibrium with aldophosphamide. These two metabolites diffuse into cells. Depending on the type of cell, in cells with low concentration of ALDH (sensitive cells as lymohocytes), aldophosphamide is spontaneously converted to phosphoramide mustard and acrolein. However, in cells with high concentration of ALDH (resistant cells such as hematopoietic stem cells) aldophosphamide is converted to carboxyphosphamide (inactive). **(B)** shows SNPs effect in Cy metabolism enzymes: SNPs in CYP450 and GSTs enzymes lead to decreased activity enzyme and produce low level of phosphoramide mustard and high level of toxic metabolites, respectively. This SNPs lead to higher incidence of GVHD, SOS and TRM.

CYP450 enzymes are important in the metabolism of drugs and xenobiotics, and gene variants as well as variability in gene expression have clinical implications. Approximately 45% of CYP-dependent drug metabolism is conducted by polymorphic CYPs, resulting in therapeutic failure and adverse reactions (4). Cy is predominantly activated by the CYP450 enzymes (CYP2C9, CYP2B6, CYP2C19 and CYP3A) (5) being metabolized to an active metabolite, 4-hydroxy-Cy, which coexists with its tautomer aldophosphamide, diffuses into cells and is spontaneously converted into the active bifunctional DNA alkylating agent (phosphoramide mustard) and acrolein, a metabolite responsible for urotoxicity (3), which has been reported to be the causative agent of hemorrhagic cystitis (HC). Detoxification of Cy metabolites occurs through phase II inactivation enzymes, aldehyde dehydrogenases (ALDH: ALDH1A1 and ALDH3A1) and glutathione S-transferases (GSTs; GSTA1, GSTM1, GSTP1 and GSTT1) (**Figure 1A**). A number of studies have indicated that resistance to Cy is determined by the activity level of cellular ALDH (6). Specifically, higher ALDH levels in hematopoietic stem cells are sufficient to induce Cy resistance in allo-HSCT (7, 8).

Genetic factors (pharmacogenetics) play a role in individual variations in the response to toxicities associated with Cy-based therapies (9). Polymorphisms in activation and detoxification genes affect both enzyme activity and metabolite levels. Several studies have identified significant pharmacogenetic factors involved in the response to Cy-based therapeutic regimens^9^. In the field of allo-HSCT, a number of publications have shown an association between gene polymorphisms in the Cy metabolism pathway and post-transplantation complications (10–14). However, these studies were carried out in HLA-matched sibling allo-HSCT with a combination of cyclosporine A and methotrexate as prophylaxis for GVHD, or in autologous transplantation. To the best of our knowledge, no such studies have been performed in the haplo-HSCT setting.

Another important drug used in the conditioning regimen for HSCT is busulfan, which is an alkylating agent generally metabolized in the liver *via* conjugation with glutathione, which is catalyzed by GST enzymes, predominantly GSTA1, GSTM1 and GSTT1. High drug exposure leads to an increased risk of sinusoidal obstruction syndrome (SOS, an early complication of hematopoietic stem cell transplantation), whereas low drug exposure has been associated with a higher risk of disease recurrence and graft failure (15). Several studies have shown that busulfan is one of the most important risk factors for SOS development (16, 17). Busulfan, particularly in combination with Cy, is associated with an increased risk of SOS (18). CYP450 enzymes play an important role in the clearance of toxic metabolites of chemotherapeutics and the glutathione pathway and are therefore involved in metabolizing busulfan and can affect the risk of SOS (19). Moreover, various studies have identified genetic factors contributing to SOS risk including GST polymorphisms such as the GSTM1-null genotype and GSTA1*B haplotype (20).

The aim of our study was therefore to identity gene polymorphisms involved in the metabolism of Cy and other drugs and correlate them with complications (GVHD, SOS, HC and TRM), after Haplo-HSCT.

## Material and methods

### Clinical cohort

We performed a retrospective cohort study of 182 consecutive patients who received a Haplo-HSCT with PT-Cy at Gregorio Marañón General University Hospital from December 2007 to June 2019. Clinical characteristics of patients are shown in **Table 1**.

**Table 1:** Clinical characteristics of 182 patients who underwent Haplo-HSCT with PT-Cy.

Characteristic	Whole cohort (n = 182)
**Recipient age (years). Median (range)**	48 (16-67)
**Donor age (years). Median (range)**	40 (14-74)
**Recipient sex (male/female)**	122/60
**Donor sex (male/female)**	100/82
**Diagnosis. n (%)**	
AML	65 (35.7)
NHL	25 (13.7)
HL	21 (11.5)
ALL	19 (10.4)
MDS	18 (9.9)
MM	5 (2.8)
Other (AA, CLL, CML)	29 (15.9)
**Status at transplant. n (%)**	
Active disease or partial response	79 (43.4)
Complete Remission	103 (56.6)
**Conditioning regimen. n (%)**	
Myeloablative	82 (45.1)
Reduced-intensity	100 (54.9)
**Previous transplant. n (%)**	64 (35.2)
Autologous transplant	46 (71.9)
Allogeneic transplant	18 (28.1)
**Cumulative incidence of aGVHD at +100 days after HSCT. n (%)**	
II-IV	71 (39)
III-IV	22 (12.1)
**Cumulative incidence of cGVHD at 2 years after HSCT. n(%)**	
Global	68 (37.4)
Moderate-severe	35 (19.2)
**Cumulative incidence of TRM at 2 years after HSCT. n (%)**	53 (29.1)
**Cumulative incidence of relapse at 2 years. n (%)**	31 (28.9)
**SOS. n (%)**	17 (9.34)
**HC. n (%)**	45 (24.73)
**OS at 5 years. n (%)**	65 (66.32)

AML, Acute myeloid leukemia; NHL, Non-Hodgkin lymphoma; HL, Hodgkin lymphoma; ALL, Acute lymphatic leukemia; MDS, Myelodysplastic syndrome; MM, Multiple myeloma; HC, Hemorrhagic cystitis; SOS, Sinusoidal obstruction syndrome; OS, Overall survival; TRM, Transplant-related mortality; AA, Aplastic anemia; CLL, Chronic lymphatic leukemia; CML, Chronic myeloid leukemia.

The local ethics committee approved the study and all recipients and donors provided written informed consent according to the Declaration of Helsinki.

The conditioning regimen for Haplo-HSCT was myeloablative (MA) for 82 patients and reduced intensity conditioning (RIC) for 100 patients. The MA conditioning regimen consisted of fludarabine 40 mg/m^2^/day from day −6 to day −3 and intravenous busulfan 3.2 mg/kg/day on either 3-4 days between days -6 to -3 or -6 to -4. The RIC regimen included fludarabine 30 mg/m^2^/day on day −6 to day −2, intravenous busulfan 3.2 mg/kg/day on either 1-2 days on days -3 and -2, and Cy (14.5 mg/kg) on day −6 and day −5. Prophylaxis against GVHD consisted of high-dose PT-Cy (50 mg/kg) administered on day +3 and day +4 post-transplantation, followed by a calcineurin inhibitor (CNI) and mycophenolate mofetil from day +5. In the absence of GVHD, mycophenolate mofetil was discontinued on day +35. CNI was withdrawn between days +60 and +90 in the absence of GVHD and discontinued by day +120.

### Samples

Genomic DNA was purified from the peripheral blood of 182 recipients pre-transplantation and from donor samples using a Maxwell RSC Blood DNA Kit (Promega, Madison, WI, USA).

Given that Cy is metabolized, primarily in the liver, to active and inactive metabolites, we employed recipient samples for this study. However, since hematopoietic stem cells show high levels of ALDH expression, we analyzed 2 SNPs in the ALDH gene in donor samples. Among the 182 haploidentical donors, we excluded four patients from the analysis due to lacking DNA samples. Thus, a total of 178 donors were analyzed.

### Posttransplant evaluation

Post-transplant complications analyzed were II-IV aGVHD, III-IV aGVHD, cGVHD, moderate-severe cGVHD, TRM, SOS and HC.

Acute GVHD was scored according to the MAGIC criteria (21). Chronic GVHD was scored according to the NIH Consensus Development Project (22). SOS was defined according to EBMT criteria (23).

### Next-generation sequencing experiments and data analysis

#### Targeted sequencing

A total of 13 genes involved in cyclophosphamide activation and detoxification *(CYP3A4, CYP3A5, CYP2A6, CYP2B6, CYP2C8, CYP2C9, CYP2C19*, *GSTA1, GSTM1, GSTP1, GSTT1, ALDH1A1* and *ALDH3A1*) were genotyped (**Supplementary Table 1**) in the recipient samples. The genotyping was performed using an enrichment-capture custom gene panel according to the manufacturer’s protocol using 50 ng of DNA. Paired-end sequencing 2x101bp was performed using the Illumina MiSeq platform. FASTQ files were aligned against the human reference genome (version GRCh37/hg19) using the Burrows Wheeler Alignment tool v0.7.9a-isis.1.01. Variant calling and indel-realignment were performed using GATK version 1.6-23-gf0210b3. The Integrative Genomics Viewer was used to visualize the variants aligned against the reference genome to confirm the variant calls by checking for possible sequencing errors.

#### Variant annotation and filtration

We employed BaseSpace software to provide the infrastructure and interface for the bioinformatics analysis. Among the total number of polymorphisms (genetic variants) analyzed, we selected those in which the presence of the minor variant (allele) is associated with the development of a complication. Variants located in the coding region and splicing sites were analyzed. We selected variants corresponding to a depth ≥30x in the canonical isoform and a variant allele frequency (VAF) ≥ 0.3 and that were represented in at least 5% of our cohort. In addition, population databases (GenomAD and 1000 genomes) were used to consult the minor allele frequencies (MAF) of each variant, to identify variants (variants with MAF ≥1%). Moreover, we used an Ensembl database to present evidence of paralogous genes of the custom panel (**Supplementary Table 2**) to ensure the veracity of the variants.

### Detection of ALDH polymorphisms in the donor samples

We selected 2 SNPs in ALDH (rs2228100 and rs887241) according to the SNPs obtained in the custom panel with previously established filters in the recipient samples (**Supplementary Table 3**). The study of ALDH SNPs was performed on the donor samples by real-time quantitative PCR using rhAmp SNP (Integrated DNA Technologies, Coralville, IA) genotyping with a LightCycler 480 System (Roche, Switzerland) according to the manufacturer’s protocol.

### Statistical analysis

Quantitative variables are expressed as median and range. Categorical variables are expressed as frequency and percentage. Fisher’s Exact Test was used to compare the distribution of categorical variables.

Cumulative incidences for univariate analysis were calculated using a Fine-Gray test from when complete remission was achieved to when an event or the last examination occurred (for those patients who did not experience an event) or when the competing risk emerged (for those patients who died or underwent transplantation without an event). The genetic variants risk of disease was assessed by calculating the subhazard ratios and their 95% confidence intervals. Probability values <0.05 were considered statistically significant.

Regression methods with competing risks were used for the multivariable analysis, including all variables with P-values <0.05 in the univariable analysis to identify possible predictors of post-haplo-HSCT complications as independent variables.

All statistical analyses were performed using SPSS v.26 (IBM Corporation, USA) and R version 3.5.1, package ‘cmprsk’.

## Results

### Gene panel features

A total of 182 recipient samples were sequenced, resulting in a median of 1,533,645 reads, The median percentage of mapped reads was 99%, and the median percentage of duplicate fragments per sample was 6.7%. The median depth coverage of the target regions was 104.9X (range 56.8X–397.3X). The sequencing data have been deposited in a public repository (NCBI SRA database) with reference PRJNA865492.

### Variant data

**Figure 2:**
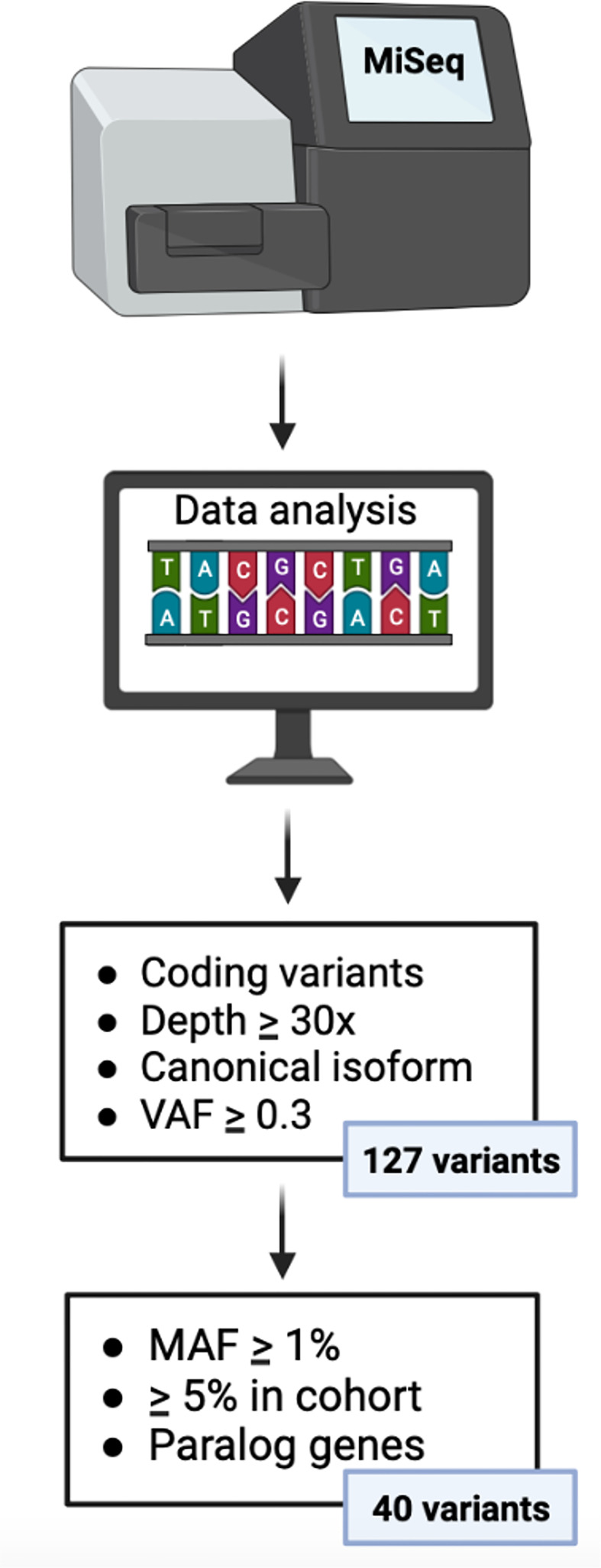
Algorithm for gene variant filtration. VAF, variant allele frequency; MAF, minor allele frequency.

Using previously defined filters, 40 variants in 11 genes of Cy metabolism were detected in the 182 recipients (**Supplementary Table 3**) (**Figure 2**). We observed 26 variants in 5 Cy activation genes (CYP450): 10 in CYP2A6, 5 in CYP2B6, 3 in CYP2C8, 4 in CYP2C9 and 4 in CYP2C19. Regarding the detoxification genes (GSTs and ALDH), we detected 2 variants in GSTA1, 3 in GSTM1, 3 in GSTP1 and 1 in GSTT1. Moreover, we detected 1 variant in ALDH1A1 and 4 in ALDH3A1. Genotype frequencies were similar to those of the 1000 Genomes Project for the Spanish population and were in accordance with the Hardy-Weinberg equilibrium, except for the frequencies of rs1065411 and rs1056806, which were in linkage disequilibrium (**Supplementary Table 3**).

The variant rs3957357 in GSTA1, included in the GSTA1*B haplotype, should not be selected according to the filters described above. Nevertheless, this variant met the criteria of depth and variant allele frequency and was represented in at least 5% of our cohort. In addition, we were able to verify the existence of this variant through the Integrative Genomics Viewer program. We therefore included the haplotype GSTA1*B in our study.

### Correlation between clinical variables and gene variants with post-transplantation complications

The post-transplantation complications analyzed were II-IV aGVHD, III-IV aGVHD, cGVHD, moderate-severe cGVHD, TRM, SOS and HC, which were not associated with clinical variables: age, recipient and donor sex, disease, status at transplant, conditioning regimen and previous transplant (**Table 2**). Report of events for polymorphisms that have been statistically significant in the univariate analysis is showed in **Supplementary Table 4**.

**Table 2:** Univariable analysis if the association between clinical variables and complications after Haplo-HSCT.

Variable	II-IV aGVHD	III-IV aGVHD	cGVHD	Mod-sev cGVHD	TRM	SOS	HC
p-value; SHR (95%CI)							
**Recipient age (≥ 48 years)**	0.66; 1.88(0.14-3.51)	0.36; 1.28 (0.54-3.07)	0.72; 0.85 (0.43-1.68)	0.11; 0.50 (0.22-1.12)	0.11; 0.87 (0.49-1.79)	0.87; 0.92 (0.56-2.45)	0.11; 0.13 (0.17-1.37)
**Donor age (≥ 40 years)**	0.63; 1.03(0.12-1.37)	0.40; 0.63 (0.33-1.59)	0.14; 1.3 (0.67-2.43)	0.09; 1.26 (0.41-2.97)	0.76; 0.77 (0.25-1.89)	0.82; 1.14 (0.72-2.34)	0.60; 1.84 (0.58-2.65)
**Recipient sex (male)**	0.41; 1.13 (0.69-2.55)	0.21; 1.66 (0.62-4.47)	0.27; 0.65 (0.31-1.34)	0.56); 1.03 (0.44-2.36)	0.55; 1.01 (0.51-2.01)	0.73; 0.89 (0.31-2.57)	0.06; 0.51(0.25-1.03)
**Donor sex (male)**	0.56; 1.00 (0.53-1.86)	0.15; 0.58 (0.24-1.38)	0.59; 0.78 (0.39-1.56)	0.31); 0.63 (0.29-1.39)	0.14; 0.60 (0.31-1.16)	0.80; 1.19 (0.43-3.27)	0.86; 0.91 (0.46-1.80)
**Underlying disease other them AML**	0.22; 0.75 (0.40-1.40)	0.20; 0.62 (0.26-1.49)	0.47; 0.74 (0.36-1.49)	0.17); 0.88 (0.71-1.09)	0.40; 0.75 (0.39-1.44)	0.61; 0.76 (0.28-2.09)	0.29; 0.66 (0.33-1.30)
**Status at transplant**							
Active disease or partial response	0.63; 1.2 (0.64-2.25)	0.17; 1.9 (0.81-4.64)	0.35; 0.82 (0.41-1.64)	0.53; 1.05 (0.47-2.31)	0.06; 1.92 (0.86-3.69)	0.56; 0.95 (0.34-2.61)	0.86; 1.12 (0.56-2.21)
**Conditioning regimen (myeloablative)**	0.16; 1.4 (0.26-1.91)	0.28; 0.6 (0.24-1.46)	0.21; 1.39 (0.70-2.75)	0.59; 0.42 (0.39-1.86)	0.47; 0.31 (0.191-2.76)	0.30; 1.18 (0.67-5.08)	0.73; 0.85 (0.43-1.69)
**Previous transplant**	0.09; 1.8 (0.93-3.48)	0.35; 1.55 (0.64-3.78)	0.51; 0.43 (0.41-1.83)	0.67; 0.80 (0.60-2.30)	0.12; 1.7 (0.88-3.33)	0.59; 1.00 (0.35-2.86)	0.27; 1.91 (0.96-3.8)

AML, Acute myeloid leukemia; aGVHD, Acute graft-versus-host disease; cGVHD, Chronic graft-versus-host disease; CI, Confidence interval; Mod-sev cGVHD, Moderate-severe cGVHD; TRM, Transplant-related mortality; SOS, Sinusoidal obstruction syndrome; SHR, Subhazard ratio; HC, Hemorrhagic cystitis.

The previously selected variants were correlated with various post-transplantation complications. Variants with statistically significant associations (p<0.05) shown in **Table 3** (univariable analysis) and **Table 4** (multivariable analysis).

**Table 3:** Univariable analysis of the association of genetic variants in cyclophosphamide metabolism genes with complications after Haplo-HSCT.

	Gene	SNP	Enzyme activity	Variant effect	Complication after Haplo-HSCT
p-value; SHR (95%CI)					
**Activation**	***CYP2A6***	rs4986892	U	Synonymous	cGVHD 0.02; 0.38 (0.12-0.98) SOS 0.03; 5.17 (1.31-6.49)
		rs1801272	Loss of function	Missense	II-IV aGVHD 0.03; 2.74 (1.20-6.25) cGVHD 0.04; 3.86 (1.07-4.06) Mod-sev cGVHD 0.0002; 7.28 (2.01-9.90)
		rs143731390	↓	Missense	TRM 0.01; 3.1 (1.13-9.48)
	***CYP2B6***	rs3745274	↓	Missense	Mod-sev cGVHD 0.01; 0.37 (0.17-0.83)
		rs3211371	↓	Missense	II-IV aGVHD 0.01; 2.46 (1.16-4.44)
		rs2279341	U	Synonymous	cGVHD 0.03; 2.11 (1.73-8.74)
		rs2279343	↑/↓	Missense	HC 0.03; 3.16 (2.8–7.2)
		rs3745274 (wt)	Normal	–	SOS 0.002; 1.6 (1.1–2.1)
	***CYP2C8***	rs10509681	↓	Missense	II-IV aGVHD 0.01; 1.59 (1.01-2.50) III-IV aGVHD 0.02; 4.07 (2.34-11.64)
		rs11572080	↓	Missense	II-IV aGVHD 0.04; 2.41 (1.91-4.68) III-IV aGVHD 0.03; 4.12 (1.04-15.91)
	***CYP2C9***	rs1799853	↓	Missense	II-IV aGVHD 0.03;1.67 (1.17-3.47) III-IV aGVHD 0.02; 4.35 (1.53-10.33)
	***CYP2C19***	rs4244285	Loss of function	Synonymous	TRM 0.01; 2.45 (1.33-8.37)
		rs3758580	U	Synonymous	TRM 0.04; 2.02 (1.14-10.87)
**Detoxification**	***GSTA1***	rs1051775	U	Synonymous	III-IV aGVHD 0.003; 0.46 (0.01-0.69)TRM 0.04; 1.86 (1.5-5.1)
		GSTA1*B	↓	Missense	III-IV aGVHD 0.01; 2.53 (1.74-10.02) TRM 0.03; 1.43 (1.3-2.8)
		(rs3957357)			
	***GSTM1***	GSTM1*0	Loss of function	Null allele	SOS 0.03; 2.3 (1.10-6.97)
	***GSTP1***	rs1695	↓	Missense	III-IV aGVHD 0.04; 2.77 (1.28-11.19)
	***GSTT1***	GSTT1*0	Loss of function	Null allele	III-IV aGVHD 0.01; 2.62 (1.179-5.86)

U, unknown SNP; CI, Confidence interval; aGVHD, acute graft-versus-host disease; cGVHD, chromic graft-versus-host disease; mod-sev cGVHD, Moderate-severe cGVHD; SOS, Sinusoidal obstruction syndrome; TRM, Transplant-related mortality; HC, Hemorrhagic cystitis; SHR, “Subhazard ratio” >1=risk; <1=protection; ↓, decreased or ↑, increased enzyme activity previously described in the bibliography; wt, wild type. CYP3A4, CYP3A5, ALDH1A1 and ALDH3A1 were studied although we did not find statistically significant differences. Decrease in enzymatic activity in the CY activation genes leads to a lower amount of the active CY metabolite. Regarding the detoxification genes, decreased activity can lead to toxic effects due to increasing levels of metabolites pathway. The enzymatic activity data were obtained from published reports (**Supplementary Table 5**).

We found no statistical significance between the variants in ALDH (recipient or donor) and post-transplantation complications.

#### Acute graft-*versus*-host disease (aGVHD)

The cumulative incidence rates for II-IV and III-IV grade aGVHD at 100 days were 39% and 12%, respectively (**Table 1**).

The variants in Cy activation genes CYP2A6*2 (rs1801272), CYP2B6*5 (rs3211371), CYP2C8 (rs10509681 and rs11572080, called CYP2C8*3 haplotype) and CYP2C9 (rs1799853) were associated with a higher incidence of grade II-IV GVHD and grade III-IV aGVHD (**Table 3**). The variants in Cy detoxification genes GSTA1*B (rs3957357), GSTP1 (rs1695) and GSTT1*0 (null allele) were correlated with a higher incidence of grade III-IV GVHD and GSTA1 (rs1051775) a lower incidence of grade III-IV GVHD (**Table 3**). In the multivariable analysis, a higher incidence of II-IV aGVHD was associated with CYP2B6*5. Moreover, GSTA1 (rs1051775) and GSTT1*0 increased the risk of III-IV aGVHD (**Table 4**).

**Table 4:** Multivariable analysis for outcomes after Haplo-HSCT with PT-Cy.

	Gene	SNP	Variant effect	Complication after Haplo-HSCT
p-value; SHR (95%CI)				
**Activation**	***CYP2A6***	rs143731390	Missense	TRM 0.003; 3.44 (1.15-9.64)
	***CYP2B6***	rs3745274	Missense	Mod-sev cGVHD 0.020; 0.38 (0.28–0.72)
		rs3211371	Missense	II-IV aGVHD 0.008; 2.02 (1.93–8.12)
**Detoxification**	***GSTA1***	rs1051775	Synonymous	III-IV aGVHD 0.042; 0.42 (0.24–0.81) TRM 0.004; 2.56 (1.37-8.47)
		GSTA1*B (rs3957357)	Missense	TRM 0.036; 2.32 (1.06-10.67)
	***GSTM1***	GSTM1*0	Null allele	SOS 0.032; 1.36 (1.11-6.32)
	***GSTT1***	GSTT1*0	Null allele	III-IV aGVHD 0.005; 3.29 (1.28-12.23)

SHR, “subhazard ratio”; CI, Confidence interval; aGVHD, acute graft-versus-host disease; cGVHD, chromic graft-versus-host disease; mod-sev, Moderate-severe; SOS, Sinusoidal obstruction syndrome; TRM, Transplant-related mortality.

#### Chronic graft-versus-host disease (cGVHD)

The cumulative incidence rates of cGVHD and moderate-severe cGVHD at two years were 37% and 19%, respectively (**Table 1**).

The variant in Cy activation gene CYP2A6 (rs1801272) was correlated with a higher incidence of cGVHD and moderate-severe cGVHD while that in CYP2B6 (rs2279341) correlated with cGVHD. In contrast, variants in CYP2A6 (rs4986892) and CYP2B6 (rs3745274) were correlated with a lower incidence of cGVHD and moderate-severe cGVHD, respectively (**Table 3**). In the multivariable analysis, only the CYP2B6*6 (rs3745274) genotype was associated with a lower incidence of moderate-severe cGVHD (**Table 4**).

#### Sinusoidal obstruction syndrome (SOS)

SOS was diagnosed in 17 patients (9%) (**Table 1**).

Two variants in activation genes and one variant in a detoxification gene were associated with a higher incidence of SOS: CYP2A6 (rs4986892), CYP2B6 (rs3745274 wild type) and GSTM1 (null allele) (**Table 3**). In the multivariable analysis, only GSTM1 maintained a statistically significant difference (**Table 4**).

Since all patients received busulfan in conditioning regimen, but doses were different, we analyzed the correlation between GSMT1*0 and SOS in the two different groups (RIC and MA). We found statistical significance between GSTM1*0 with higher risk of SOS in patients with MA conditioning regimen [p-value 0.04; SHR (3.22); 95% CI (1.01-5.24)], but not for patients with RIC conditioning regimen [p-value 0.33; SHR (0.45); 95% CI (0.09-2.28)].

#### Hemorrhagic cystitis (HC)

Forty-five patients presented HC (25%) (**Table 1**).

The carriers of CYP2B6*4 (rs2279343) had a significantly higher incidence of HC (**Table 3**), but there were no statistically significant differences in the multivariable analysis (**Table 4**).

#### Transplant-related mortality (TRM)

The cumulative incidence for TRM at two years was 29% (**Table 1**).

Variants in CYP2A6*35 (rs143731390), CYP2C19*2 (rs4244285, rs3758580) were associated with an increased risk of TRM. Regarding the enzymes involved in detoxification, we found that the GSTA1*B haplotype increased the risk of TRM (**Table 3**). In the multivariable analysis, our results showed that CYP2A6*35 (rs143731390) and GSTA1*B were correlated with an increased risk of TRM, and GSTA1 rs1051775 was correlated with a decreased risk of TRM (**Table 4**).

## Discussion

Several gene variants have been associated with changes in the metabolism or the effect of drugs (24), which could explain interindividual variability in drug responses and toxicities. In allo-HSCT, polymorphisms involved in the metabolism of drugs (Cy, busulfan, melphalan, methotrexate, cyclosporine A and tacrolimus) used in conditioning regimens or GVHD prophylaxis have been related to post-HSCT complications (10). To the best of our knowledge, however, the role of polymorphisms in Cy metabolism genes in the setting of unmanipulated haplo-HSCT with high dose PT-Cy has not been described. Cy is an inactive prodrug whose bioactivation requires the activity of phase I CYP450 enzymes metabolism in the liver. Detoxification of Cy metabolites occurs by phase II inactivation enzymes, aldehyde dehydrogenases and glutathione S-transferases. In this context, a number of gene variants have been associated with a loss of CYP450 enzyme activity. Variants in detoxification genes also lead to a decreased ability to eliminate Cy metabolites due to lowered enzyme activity, which leads to increased amounts of toxic metabolites. Thus, variants in GST genes often result in impaired detoxification (**Figure 1B**).

### Graft-*versus*-host-disease (GVHD)

Our study found that 6 variants previously reported to produce lower levels of the activation enzymes CYP2A6, CYP2B6, CYP2C8 and CYP2C9 were significantly correlated with the development of both aGVHD and cGVHD. Lower Cy production would result in lesser amounts of alkylating agent (phosphoramide mustard), which is responsible for eliminating alloreactive T cells (1) (**Figure 1A**). To the best of our knowledge, no previous studies have correlated the variants in these enzymes with a higher probability of developing GVHD. CYP2A6 is an enzyme with a minor role in Cy metabolism. The variant allele CYP2A6*2 (rs1801272), which does not produce functional enzyme (25), was associated with a higher risk of grade II-IV aGVHD, cGVHD and moderate-severe cGVHD. The rs4986892 variant of CYP2A6, whose function has not been described, was correlated with cGVHD in our study. CYP2B6 is the major activation enzyme of Cy in the liver (2), and genetic polymorphisms of this enzyme contribute to interindividual variations in CYP2B6 activity and expression (26, 27). CYP2B6*5 (rs3211371) is associated with reduced enzyme production (14, 28) and 50% less 4-hydroxycyclophosphamide compared with the wild-type (14). In our study, this variant correlated with a high incidence of II-IV aGVHD in the multivariable analysis. We also found that polymorphism rs2279341 in the CYP2B6 gene was associated with cGVHD; however, there is no reported information about the effect of this synonymous variant on protein production or function. The variant CYP2B6*6 (rs3745274) was also found to be associated with a lower incidence of moderate-severe cGVHD in the univariable and multivariable analyses. This association might be due to the fact that the variant allele leads to a decreased protein expression, although the protein has an enhanced catalytic ability (29).

Two variants in the CYP2C8 gene (rs11572080 and rs10509681, denoted as CYP2C8*3), which has a minor role in Cy metabolism, were correlated with grades II-IV and III-IV aGVHD. These two polymorphisms have been shown to have decreased CYP2C8 activity in substrates such as the anticancer drug paclitaxel (30). However, no studies have correlated these variants with GVHD so far. Another enzyme highly involved in Cy metabolism is CYP2C9 (5). We observed a significant association between the presence of CYP2C9*2 (rs1799853) and II-IV and III-IV aGVHD. This variant has already been reported to have lower 4-hydroxylase activity (5), than the wild type.

Detoxification of Cy metabolites mainly occurs through various ALDH enzymes (ALDH1A1 and ALDH3A1), as well as through various GST enzymes (GSTA1, GSTM1, GSTP1 and GSTT1). GSTA1 is the primary GST in the liver and is important for the detoxification of Cy and busulfan (9). GSTA1 has 3 linked polymorphisms in the promoter region, and 2 genotypes (GSTA1^∗^A and GSTA1^∗^B) have been defined based on the linked polymorphisms −631 T>G, −567 T>G, −69 C>T (rs3957357), and −52 G>A. The GSTA1∗B haplotype (31) has been associated with reduced protein levels (32) and therefore reduced detoxification and increased exposure to activated Cy metabolites (33). Moreover, GSTA1 is the main enzyme involved in busulfan metabolism. Previous studies have reported that GSTA1∗B significantly reduced busulfan clearance (34, 35). In our study, we found a correlation between GSTA1*B and a higher incidence of III-IV aGVHD. The decreased activity of the enzyme could therefore lead to an accumulation of toxic metabolites. Cy is not directly toxic to the sinusoidal endothelial cells; however, the phosphoramide mustard, which is a substrate for GSTA1, causes cellular toxicity (36). Indeed, GVHD is a complication related to tissue damage that occurs based on the conditioning regimen and the resulting inflammatory cytokines (37). Previous studies showed that patients with GSTA1*A (a variant with higher enzymatic activity than GSTA1*B) have a lower incidence of GVHD, because less tissue damage occurs, a finding consistent with our study (13). Another variant associated with III-IV aGVHD is rs1051775 in the GSTA1 gene. However, this is a synonymous variant, and there have been no studies reporting its effect on the protein. GSTP01 encodes for an enzyme with significant affinity for Cy metabolites. The single-nucleotide substitution 313 A>G (rs1695) results in an amino acid change associated with lower substrate-specific catalytic activity and therefore a slower elimination of toxic metabolites. A number of studies have shown that patients with rs1695 present with increased toxicity when treated with Cy (9). In our study, patients carrying rs1695 had an increased risk of developing III-IV aGVHD, which is consistent with previous studies correlating rs1695 with a higher incidence of GVHD (10). Polymorphic deletions in GSTT1 are common in the general population (38). In our study we found a correlation between GSTT1*0 (null allele), which leads to increased amounts of toxic metabolites, and a higher incidence of III-IV aGVHD (also in the multivariable analysis).

### Sinusoidal obstruction syndrome (SOS)

Three variants in 3 genes (*CYP2A6, CYP2B6 and GSTM1)* were significantly correlated in our study with a higher incidence of SOS. The CYP2B6*6 has a dual effect, since the variant allele leads to decreased gene expression, although the resulting protein has an enhanced catalytic ability. Carriers of the wild-type genotype are therefore supposed to have higher enzymatic activity and to be better producers of active cyclophosphamide metabolites (29). Our study found that the incidence of SOS was higher in carriers of the GG genotype CYP2B6*6 rs3745274 (wild type), which is consistent with previous studies (10). We also found a correlation between GSTM1*0 (null allele) and the incidence of SOS, which remained statistically significant in the multivariable study. These results were previously reported in allo-HSCT (36). Moreover, busulfan cause hepatic toxicity that might be worsened by Cy exposure, resulting in SOS (39). These results confirm that the GSTM1*0 genotype is an important risk factor for SOS, specifically for patients receiving an MA conditioning regimen, and its testing should be implemented in routine laboratories to design effective strategies to prevent this serious complication.

### Hemorrhagic cystitis (HC)

We studied the correlation between gene polymorphisms in Cy metabolism and HC and found that the variant CYP2B6*4 (rs2279343) was associated with HC. Previous reports had correlated this variant with a higher incidence of oral mucositis (10). Different studies have shown that this variant produces either an increase or a decrease in CYP2B6*4 activity (29, 40), therefore more information is needed in order to better understand the biological basis of its association with the development of HC.

### Transplant-related mortality (TRM)

Although the complications and mortality associated with transplantation have decreased in recent years, TRM is still the major barrier to allo-HSCT. There are studies that have shown that increased exposure to toxic Cy metabolites leads to increased liver toxicity and mortality (41). Three variants in 2 activation enzymes *(CYP2A6, CYP2C19)* were correlated with a higher probability of TRM. The missense variant in CYP2A6 (rs143731390), which leads to decreased enzyme activity and therefore greater toxicity (42), was significantly associated with TRM and remained so in the multivariable analysis. Regarding the CYP2C19 enzyme, two synonymous variants (rs4244285 and rs3758580) were significantly related to TRM. Variant rs4244285 produces an aberrant splice site altering the reading frame and leading to a premature stop codon and ultimately to a nonfunctional protein (43). This SNP was previously reported in a study showing that the CYP2C19*2 genotype had an impact on TRM (11). Patients classified as poor metabolizers had significantly higher hepatotoxicity and nephrotoxicity and greater side effects, all of which can affect TRM (11). With regard to detoxification enzymes, our study found correlation between 2 variants in *GSTA1* and higher TRM in the univariable and multivariable analyses. The rs1051775 and GSTA1*B variants have been associated with reduced protein levels, causing poor drug metabolism, thereby possibly leading to higher cell toxicity.

In conclusion, we found significant correlations between variants in genes related to Cy metabolism and GVHD and other toxicity related complications after Haplo-HSCT. Overall, variants associated with a decreased activity of enzymes that activate Cy (low levels of active metabolites) were correlated with increased aGVHD, cGVHD, TRM and SOS. In contrast, variant associated with low activity of detoxification enzymes (high levels of toxic metabolites) were correlated with a higher incidence of severe GVHD, TRM and SOS.

Although the clinical relevance of these polymorphisms should be validated in other cohorts and other types of allo-HSCT with PT-Cy, testing for these variants before transplantation could facilitate personalized risk assessment and improve the clinical management of patients undergoing Haplo-HSCT.

## Data availability statement

The data presented in the study are deposited in the NCBI SRA repository, accession number PRJNA865492.

## Ethics statement

The studies involving human participants were reviewed and approved by the Ethics Committee of the Gregorio Marañón General University Hospital. The patients/participants provided their written informed consent to participate in this study.

## Author contributions

PM, CA-Z,CM-L,and IB, were responsible for conception and design. RB, IGC, ND, JA, MK, and JD-M provided patients and managed samples.PM, CA-Z, DC, MC, DG,JS-G, CM-L,and IB collected and assembled data. PM, CA-Z, CM-L, and IB were responsible for data analysis and interpretation. PM, CM-L, and IB wrote the manuscript. All authors gave final approval ofthe manuscript and are accountable for all aspects of the work.

## Funding

This study was partially supported by Ministry of Economy and Competitiveness ISCIII-FIS Grants PI17/01880 and PI20/00521 and cofinanced by the European Regional Development Fund from the European Commission, the “A way of making Europe” initiative.

## Acknowledgments

The authors wish to acknowledge the staff of the Hematology Department, as well as the patients and their families.

## Conflict of interest

The authors declare that the research was conducted in the absence of any commercial or financial relationships that could be construed as a potential conflict of interest.

## Publisher’s note

All claims expressed in this article are solely those of the authors and do not necessarily represent those of their affiliated organizations, or those of the publisher, the editors and the reviewers. Any product that may be evaluated in this article, or claim that may be made by its manufacturer, is not guaranteed or endorsed by the publisher.
