# Supplementary material for: Association between gene polymorphisms in the cyclophosphamide metabolism pathway with complications after haploidentical hematopoietic stem cell transplantation
Source: Front Immunol. 2022 Sep 23;13:1002959. doi: 10.3389/fimmu.2022.1002959 (PMC9537744; doi:10.3389/fimmu.2022.1002959)
Supplement: Supplementary file 1 [file DataSheet_1.pdf]

Gene	Transcript ID
ALDH1A1	ENST00000297785.3
ALDH3A1	ENST00000457500.2
CYP2A6	ENST00000301141.5
CYP2B6	ENST00000324071.4
CYP2C19	ENST00000371321.3
CYP2C8	ENST00000371270.3
CYP2C9	ENST00000260682.6
CYP3A4	ENST00000336411.2
CYP3A5	ENST00000222982.4
GSTA1	ENST00000334575.5
GSTM1	ENST00000309851.5
GSTP1	ENST00000398606.3
GSTT1	ENST00000248935.5

Supplementary Table 1. Genes included in the *custom* panel of cyclophosphamide activation and detoxification genes. The coding regions of the targeted genes were studied.
