# Supplementary material for: Association between gene polymorphisms in the cyclophosphamide metabolism pathway with complications after haploidentical hematopoietic stem cell transplantation
Source: Front Immunol. 2022 Sep 23;13:1002959. doi: 10.3389/fimmu.2022.1002959 (PMC9537744; doi:10.3389/fimmu.2022.1002959)
Supplement: Supplementary file 2 [file DataSheet_2.pdf]

**Gene**	**Paralog genes**															
ALDH1A1	*ALDH1A2*	*ALDH1A3*	*ALDH2*	*ALDH1B1*	*ALDH1L1*	*ALDH1L2*	*ALDH9A1*	*ALDH8A1*	*ALDH5A1*	*ALDH7A1*	*ALDH6A1*	*ALDH4A1*	*ALDH16A1*	*ALDH3A1*	*ALDH3A2*	*ALDH3B1*
ALDH3A1	*ALDH3A2*	*ALDH3B1*	*ALDH3B2*	*ALDH1A3*	*ALDH1A1*	*ALDH1A2*	*ALDH2*	*ALDH1B1*	*ALDH8A1*	*ALDH1L1*	*ALDH9A1*	*ALDH1L2*	*ALDH5A1*	*ALDH4A1*	*ALDH6A1*	*ALDH7A1*
CYP2A6	*CYP2A13*	*CYP2A7*	*CYP2F1*	*CYP2B6*	*CYP2C19*	*CYP2S1*	*CYP2C18*	*CYP2C9*	*CYP2C8*	*CYP2E1*	*CYP2D6*	*CYP2U1*	*CYP2J2*	*CYP2D7*	*CYP2W1*	*CYP2R1*
CYP2B6	*CYP2A13*	*CYP2A7*	*CYP2A6*	*CYP2C8*	*CYP2C9*	*CYP2S1*	*CYP2F1*	*CYP2C19*	*CYP2C18*	*CYP2E1*	*CYP2D6*	*CYP2U1*	*CYP2J2*	*CYP2D7*	*CYP2W1*	*CYP2R1*
CYP2C19	*CYP2C9*	*CYP2C18*	*CYP2C8*	*CYP2E1*	*CYP2A13*	*CYP2F1*	*CYP2A6*	*CYP2B6*	*CYP2A7*	*CYP2S1*	*CYP2D6*	*CYP2U1*	*CYP2J2*	*CYP2D7*	*CYP2W1*	*CYP2R1*
CYP2C8	*CYP2C19*	*CYP2C18*	*CYP2C9*	*CYP2E1*	*CYP2A13*	*CYP2F1*	*CYP2A6*	*CYP2B6*	*CYP2A7*	*CYP2S1*	*CYP2D6*	*CYP2U1*	*CYP2J2*	*CYP2D7*	*CYP2W1*	*CYP2R1*
CYP2C9	*CYP2C19*	*CYP2C18*	*CYP2C8*	*CYP2E1*	*CYP2A13*	*CYP2F1*	*CYP2A6*	*CYP2B6*	*CYP2A7*	*CYP2S1*	*CYP2D6*	*CYP2U1*	*CYP2J2*	*CYP2D7*	*CYP2W1*	*CYP2R1*
CYP3A4	*CYP3A7*	*CYP3A7-CYP3A51P*	*CYP3A5*	*CYP3A43*												
CYP3A5	*CYP3A7*	*CYP3A7-CYP3A51P*	*CYP3A4*	*CYP3A43*												
GSTA1	*GSTA2*	*GSTA5*	*GSTA3*	*GSTA4*	*GSTP1*	*GSTM2*	*GSTM1*	*GSTM4*	*GSTM3*	*GSTM5*	*HPGDS*					
GSTM1	*GSTA2*	*GSTM4*	*GSTM2*	*GSTA4*	*GSTM3*	*GSTA1*	*GSTA5*	*GSTA3*	*GSTP1*	*GSTM5*	*HPGDS*					
GSTT1	No data for paralogs															
GSTP1	*GSTA2*	*GSTA1*	*GSTA3*	*GSTA4*	*GSTA5*	*GSTM3*	*GSTM4*	*GSTM2*	*GSTM1*	*GSTM5*	*HPGDS*					

Supplementary Table 2. Paralog genes for each gene of the custom panel. Paralog relationships in the hg19 reference genome were obtained from ENSEMBL
