# Supplementary material for: Association between gene polymorphisms in the cyclophosphamide metabolism pathway with complications after haploidentical hematopoietic stem cell transplantation
Source: Front Immunol. 2022 Sep 23;13:1002959. doi: 10.3389/fimmu.2022.1002959 (PMC9537744; doi:10.3389/fimmu.2022.1002959)
Supplement: Supplementary file 4 [file DataSheet_3.pdf]

Gene	SNP	aGVHD II-IV (n=75)	aGVHD III-IV (n=24)	cGVHD (n=64)	mod-sev cGVHD (n=34)	TRM (n=52)	SOS (n=17)	HC (n=45)
**CYP2A6**	rs4986892	-	-	n=10	-	-	n=11	-
	rs1801272	n=10	-	n=9	n=8	-	-	-
	rs143731390	-	-	-	-	n=8	-	-
**CYP2B6**	rs3745274	-	-	-	n=10	-	-	-
	rs3211371	n=12	-	-	-	-	-	-
	rs2279341	-	-	n=8	-	-	-	-
	rs2279343	-	-	-	-	-	-	n=6
	rs3745274 (wt)	-	-	-	-	-	n=8	-
**CYP2C8**	rs10509681	n=32	n=17	-	-	-	-	-
	rs11572080	n=32	n=17	-	-	-	-	-
**CYP2C9**	rs1799853	n=17	n=8	-	-	-	-	-
**CYP2C19**	rs4244285	-	-	-	-	n=13	-	-
	rs3758580	-	-	-	-	n=11	-	-
**GSTA1**	rs1051775	-	n=12	-	-	n=25	-	-
	GSTA1*B	-	n=19	-	-	n=22	-	-
**GSTM1**	GSTM1*0	-	-	-	-	-	n=15	-
**GSTP1**	rs1695	-	n=18	-	-	-	-	-
**GSTT1**	GSTT1*0	-	n=10	-	-	-	-	-

Supplementary Table 4. Number of events for each polimorphism that has been statistically significant in the univariate analysis. aGVHD: acute graft-versus-host disease; cGVHD: chromic graft-versus-host disease; mod-sev cGVHD: Moderate-severe cGVHD; SOS: Sinusoidal obstruction syndrome; TRM: Transplant-related mortality; HC: Hemorrhagic cistitis. Symbol "-" indicates polymorphisms that are not statistically significant for that post-transplant complication. Polymorphisms (genetic variant) selected included the presence of the minority variant, taking into account heterozygotes and homozygotes.
