# Supplementary material for: Association between gene polymorphisms in the cyclophosphamide metabolism pathway with complications after haploidentical hematopoietic stem cell transplantation
Source: Front Immunol. 2022 Sep 23;13:1002959. doi: 10.3389/fimmu.2022.1002959 (PMC9537744; doi:10.3389/fimmu.2022.1002959)
Supplement: Supplementary file 5 [file DataSheet_4.pdf]

Gene	SNP	Enzyme activity	Variant effect	Reference
CYP2A6	rs4986892	U	Synonymous	
	rs1801272	Loss of function	Missense	[1]
	rs143731390	↓	Missense	[2]
CYP2B6	rs3745274	↓	Missense	[3]
	rs3211371	↓	Missense	[4,5]
	rs2279341	U	Synonymous	
	rs2279343	↑/↓	Missense	[6,7]
	rs3745274 (wt)	Normal	-	[3]
CYP2C8	rs10509681	↓	Missense	[8]
	rs11572080	↓	Missense	
CYP2C9	rs1799853	↓	Missense	[9]
CYP2C19	rs4244285	Loss of function	Synonymous	[10]
	rs3758580	U	Synonymous	
GSTA1	rs1051775	U	Synonymous	
	GSTA1*B (rs3957357)	↓	Missense	[11]
GSTM1	GSTM1*0	Loss of function	Null allele	[12]
GSTP1	rs1695	↓	Missense	[12]
GSTT1	GSTT1*0	Loss of function	Null allele	[12]

Supplementary Table 5. Effect on the enzymatic activity of the genetic variants in cyclophosphamide metabolism genes and their association with complications after Haplo-HSCT. U: unknown; ↓: decreased or ↑: increased enzyme activity previously described in the bibliography; WT: wild type.

[1] Di YM, Chow VD, Yang LP, Zhou SF. Structure, function, regulation and polymorphism of human cytochrome P450 2A6. Curr Drug Metab. 2009;10(7):754-780.

[2] Al Koudsi N, Ahluwalia JS, Lin SK, Sellers EM, Tyndale RF. A novel CYP2A6 allele (CYP2A6*35) resulting in an amino-acid substitution (Asn438Tyr) is associated with lower CYP2A6 activity in vivo. Pharmacogenomics J. 2009;9(4):274-282.

[3] Rocha V, Porcher R, Fernandes JF, et al. Association of drug metabolism gene polymorphisms with toxicities, graft-versus-host disease and survival after HLA-identical sibling hematopoietic stem cell transplantation for patients with leukemia. Leukemia. 2009;23(3):545-556.

[4] Zanger UM, Klein K. Pharmacogenetics of cytochrome P450 2B6 (CYP2B6): advances on polymorphisms, mechanisms, and clinical relevance. Front Genet. 2013;4:24. Published 2013 Mar 5.

[5] Bachanova V, Shanley R, Malik F, et al. Cytochrome P450 2B6*5 Increases Relapse after Cyclophosphamide-Containing Conditioning and Autologous Transplantation for Lymphoma. Biol Blood Marrow Transplant. 2015;21(5):944-948.

[6] Labib RM, A Abdelrahim ME, Elnadi E, Hesham RM, Yassin D. CYP2B6rs2279343 Is Associated with Improved Survival of Pediatric Rhabdomyosarcoma Treated with Cyclophosphamide. PLoS One. 2016;11(7):e0158890. Published 2016 Jul 7.

[7] Xie HJ, Yasar U, Lundgren S, et al. Role of polymorphic human CYP2B6 in cyclophosphamide bioactivation. Pharmacogenomics J. 2003;3(1):53-61.

[8] Dai D, Zeldin DC, Blaisdell JA, et al. Polymorphisms in human CYP2C8 decrease metabolism of the anticancer drug paclitaxel and arachidonic acid. Pharmacogenetics. 2001;11(7):597-607.

[9] Timm R, Kaiser R, Lötsch J, et al. Association of cyclophosphamide pharmacokinetics to polymorphic cytochrome P450 2C19. Pharmacogenomics J. 2005;5(6):365-373.

[10] Hirota T, Eguchi S, Ieiri I. Impact of genetic polymorphisms in CYP2C9 and CYP2C19 on the pharmacokinetics of clinically used drugs. Drug Metab Pharmacokinet. 2013;28(1):28-37.

[11] Guy CA, Hoogendoorn B, Smith SK, Coleman S, O'Donovan MC, Buckland PR. Promoter polymorphisms in glutathione-S-transferase genes affect transcription. Pharmacogenetics. 2004;14(1):45-51.

[12] Hayes JD, Strange RC. Glutathione S-transferase polymorphisms and their biological consequences. Pharmacology. 2000;61(3):154-166.
